# Poly(vinyl Chloride) Photostabilization in the Presence of Schiff Bases Containing a Thiadiazole Moiety

**DOI:** 10.3390/molecules23040913

**Published:** 2018-04-15

**Authors:** Naser Shaalan, Nawres‎‏ Laftah, Gamal A. El-Hiti, Mohammad Hayal Alotaibi, Raad Muslih, Dina S. Ahmed, Emad Yousif

**Affiliations:** 1 Department of Chemistry, College of Science for Women, University of Baghdad, Baghdad 10071, Iraq; ndsh1972@gmail.com (N.S.); nawrw.a200@gmail.com (N.L.); raadmuslihr@gmail.com (R.M.); 2 Cornea Research Chair, Department of Optometry, College of Applied Medical Sciences, King Saud University, P.O. Box 10219, Riyadh 11433, Saudi Arabia; 3 Center of Excellence in Integrated Nano-Systems, King Abdulaziz City for Science and Technology, P.O. Box 6086, Riyadh 11442, Saudi Arabia; mhhalotaibi@kacst.edu.sa; 4 Department of Chemistry, College of Science, Tikrit University, Tikrit 34001, Iraq; dinasaadi86@gmail.com; 5 Department of Chemistry, College of Science, Al-Nahrain University, Baghdad 64021, Iraq

**Keywords:** poly(vinyl chloride), photostability, Schiff bases, thiadiazole, average molecular weight, quantum yield, irradiation

## Abstract

Five Schiff bases containing a thiadiazole moiety have been used as poly(vinyl chloride) photostabilizers at low concentrations. The efficiency of Schiff bases as photostabilizers was investigated using various techniques, for example, the changes in poly(vinyl chloride) infrared spectra, molecular weight, chain scission quantum yield, and surface morphology were monitored upon irradiation with an ultraviolet light. Evidently, all the additives used inhibited poly(vinyl chloride) photodegradation at a significant level. The most efficient Schiff base exhibited a high level of aromaticity and contained a hydroxyl group. It seems possible that such photostabilization could be due to the direct absorption of ultraviolet radiation by the additives. In addition, Schiff bases could act as radical scavengers and proton transfer facilitators to stabilize the polymeric materials.

## 1. Introduction

The global production and consumption of plastics have increased over the years [1]. Poly(vinyl chloride) (PVC) is ranked third among the most produced plastics, next to polyethylene and polypropylene [2]. The production cost of PVC is low and involves polymerization of vinyl chloride [3,4,5]. The majority (80%) of large-particle (100–180 μm diameters) PVC is produced through suspension polymerization [6]. The other 20% of PVC is produced through emulsion (12%) and bulk (8%) polymerization with an average size of ca. 0.2 μm [6]. PVC properties are completely different from those of polyethylene, due to the presence of chlorine. The chlorine percentage amounted to 57% of the PVC mass [7]. PVC is normally linear, rigid, strong, and white in color. PVC melts at ca. 170–180 °C, leading to its decomposition, discoloration, and the liberation of hydrogen chloride [8,9,10,11]. In addition, long-term exposure of PVC to sunlight and high temperature could lead to the loss of mechanical properties and surface roughness [12,13,14]. The rigidity and photodegradation of PVC are of major concern, therefore, PVC has to be softened and stabilized against irradiation to be used in various applications [15].

**Figure 1:**
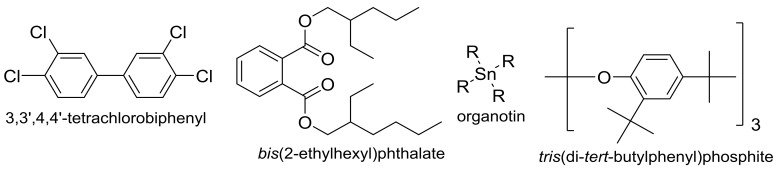
Some common PVC additives.

Additives need to be incorporated into PVC so it can be used commercially. Commercial additives include ultraviolet (UV) and heat stabilizers, thermal and impact modifiers, blowing reagents, flame retardants, and smoke suppressors [16]. Various factors should be taken into consideration with respect to the choice of additives. For example, the color, compatibility, and volatility of additives, cost, requirements for regularity approval and performance of modified PVC are the most important factors [16]. Polychlorinated biphenyls (e.g., 3,3′,4,4′-tetrachlorobiphenyl; Figure 1) were added to PVC as stabilizers and flame retardants in the past. However, polychlorinated biphenyls have been proven to be carcinogens and have bad impact on the environment. They have therefore been banned [17]. Phthalic acids, such as *bis*(2-ethylhexyl) phthalate (Figure 1), have been used as plasticizers as they are cheap, mostly oil, have low volatility, and mix well with PVC [16]. However, phthalates are under pressure in Europe due to the potential risk associated with their uses in medical applications, such as blood bags. Metal stabilizers, such as cadmium, lead, and tin-based stabilizers (Figure 1) are very common. Additionally, various phosphite stabilizers (e.g., *tris*(di-*tert*-butylphenyl) phosphite; Figure 1) have been commercially used. Barium-zinc stabilizer mixtures have no specific hazards and has been used as a replacement for cadmium-based ones [18]. However, for optimum PVC performance, barium-zinc stabilizers require the use of co-stabilizers [18,19,20]. Therefore, research has been developed to synthesize new PVC additives.

Recent research shows that various additives have been used successfully at low concentrations to reduce PVC photodegradation. The most interesting additives include metal complexes [21,22,23,24], Schiff bases [25,26,27,28], organic compounds [29,30,31] and many others [32,33,34,35,36,37]. Various additives that contain organic-inorganic sheets have been used in perovskite solar cell devices as energy sources [38,39,40,41,42]. Now, we report the effective use of Schiff’s bases containing thiadiazole ring systems at low concentrations, as PVC additives, to inhibit its photodegradation as part of our continuing research in the area of polymers [43,44,45,46,47,48,49,50]. Schiff bases are electron rich and act as UV absorbers. Therefore, Schiff bases containing a thiadiazole moiety are expected to absorb UV irradiation and protect PVC against photodegradation.

## 2. Results and Discussions

### 2.1. Schiff Bases ***1**–**5***

**Figure 2:**
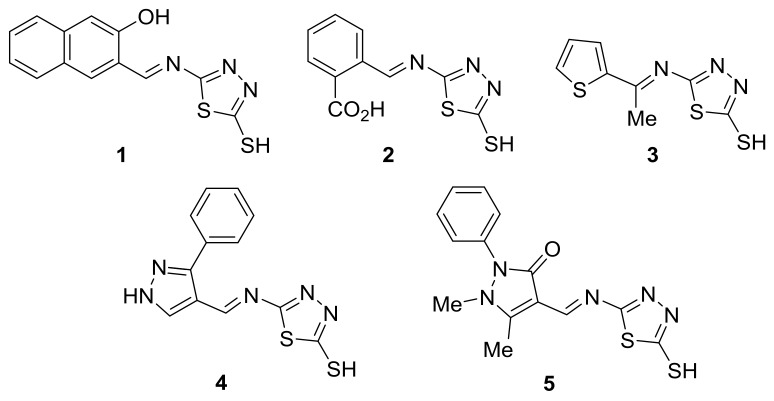
Schiff bases **1**–**5**.

Schiff bases **1**–**5** (Figure 2) were obtained based on a procedure from the literature [51]. Reaction of an equimolar mixture of 5-amino-1,3,4-thiadiazole-2-thiol and a number of carbonyl compounds in anhydrous ethanol in acidic medium (acetic acid) under reflux for 2.5 to 4 h gave the crude products. Crystallization of the crude product using ethanol gave pure Schiff bases **1**–**5** in 79–88% yields [51]. The color of Schiff bases **1**–**5** ranged from colorless to orange.

The structures of Schiff bases **1**–**5** were established using IR, UV, ^1^H-NMR, and mass spectroscopy (Table 1, Table 2 and Table 3). The IR spectra of Schiff bases **1–5** showed the presence of the CH=N bonds as an intense signals at 1597–1618 cm^–1^. In addition, the SH bonds appeared at 2564–2604 cm^–1^ (Table 1). For Schiff bases **2** and **5**, the C=O bonds appeared as strong bands at 1786 and 1685 cm^–1^, respectively. The OH bonds in Schiff bases **1** and **2** resonated as broad bands at 3318 and 3124 cm^–1^, respectively. The UV spectra of Schiff bases **1**–**5** showed absorption bands due to both π–π* (274–331 nm) and n–π* (383–484 nm) electronic transitions.

The ^1^H-NMR spectra for Schiff bases **1**–**5** showed exchangeable singlets at 12.31–14.30 ppm corresponding to the SH protons. Moreover, they showed characteristic singlets at 7.91–9.90 ppm corresponding to the CH=N protons for Schiff bases **1**, **2**, **4**, and **5** (Table 2). It should be noted that the OH proton in **1** and the CO_2_H proton in **2** were not seen, but was expected [52]. Mass spectra showed the molecular ion peaks for Schiff bases **1**–**5** (Table 3).

### 2.2. PVC Photodegradation by Fourier Transform Infrared (FTIR) Spectroscopy

**Figure 3:**
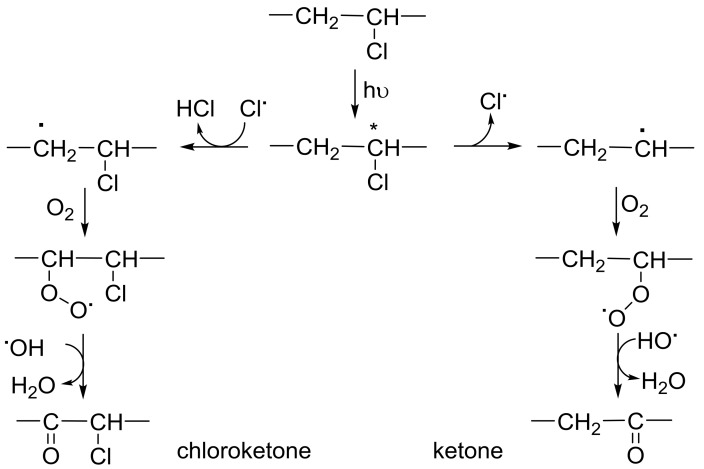
Photo-oxidation of PVC.

**Figure 4:**
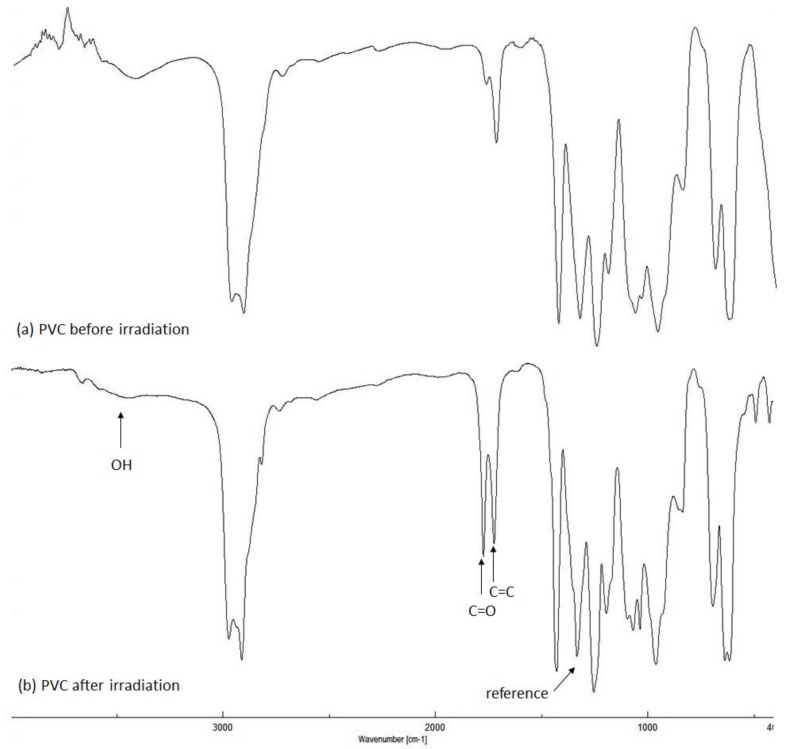
FTIR spectra for PVC.

PVC photo-oxidation leads to the production of various fragments [53]. Ketones and chloroketeones (C=O) are the most abundant ones (Figure 3). In addition, alkene (C=C) and alcohol (OH) moieties are produced through the elimination of hydrogen chloride and peroxidation of PVC, respectively [29]. However, the abundance of such functional groups are lower compared to those for ketone fragments [29]. Indeed, the changes in C=O and C=C and OH signal intensities in FTIR spectra of PVC after irradiation were very noticeable compared to those obtained before irradiation (Figure 4).

**Figure 5:**
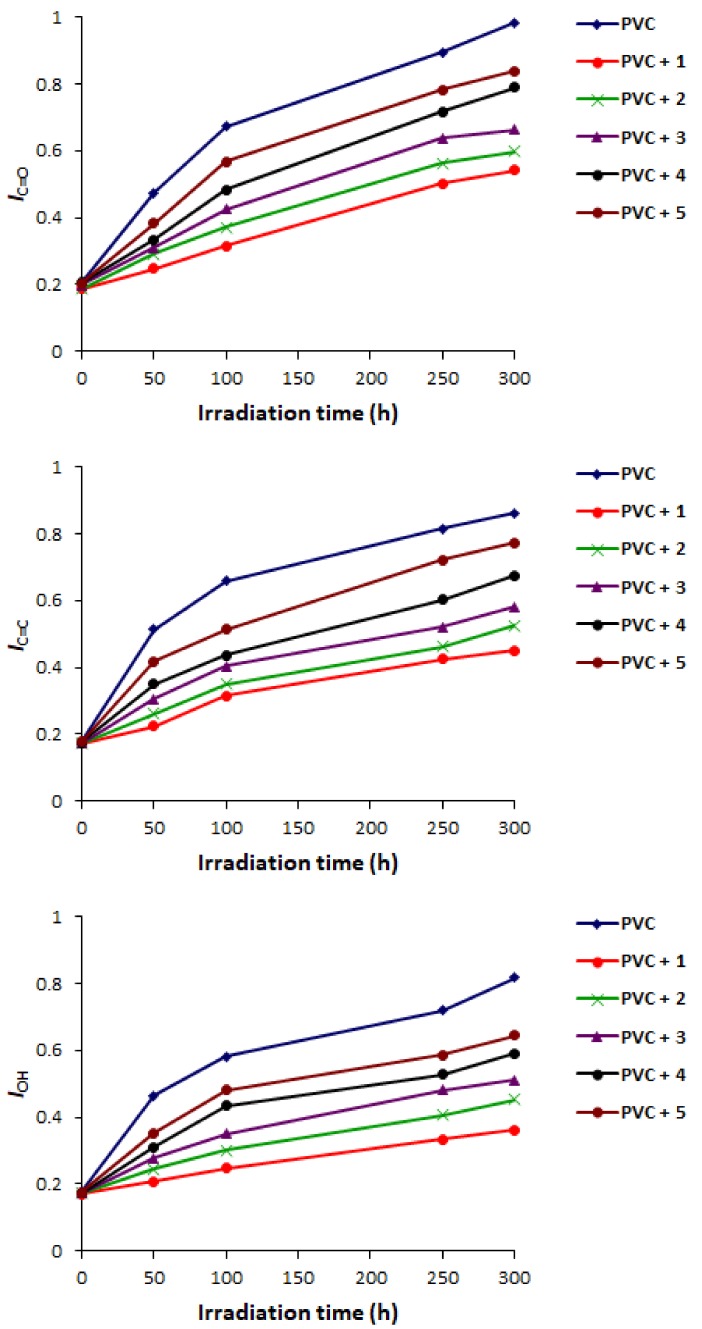
Changes in *I*_C=O_, *I*_C=C_, and *I*_OH_ for PVC.

A mixture of Schiff bases **1**–**5** at the concentration of 0.5% by weight and PVC in tetrahydrofuran (THF) was stirred to ensure complete homogeneity. Various films were produced to investigate the role of additives **1**–**5** on the photostabilization of the polymeric materials. The films were exposed to UV irradiation for 300 h and the changes in absorption bands noticed at 1724 (C=O), 1631 (C=C), and 3400 cm^–1^ (OH) were checked. The changes in such functional group intensities were compared to the peak appeared 1328 cm^–1^ attributed to the CH_2_ bonds within polymer backbone [54]. The indices of C=O (*I*_C=O_), C=C (*I*_C=C_), and OH (*I*_OH_) groups were measured from the IR spectra. Evidently, the changes in *I*_C=O_, *I*_C=C_, and *I*_OH_ were very high for the blank PVC films compared to the films containing the additives (Figure 5). Schiff base **1**, which contains 2-naphthaol moiety, was the most efficient additive in inhibiting PVC photodegradation. The hydroxyl group within the structure of **1** stabilized the PVC since it acts as a radical scavenger [55]. Additionally, it facilitates the internal conversion, proton transfer and intersystem conversion between the excited and ground states within PVC and, therefore, inhibits polymeric materials photodegradation [56].

### 2.3. PVC Photodegradation by Viscosity

**Figure 6:**
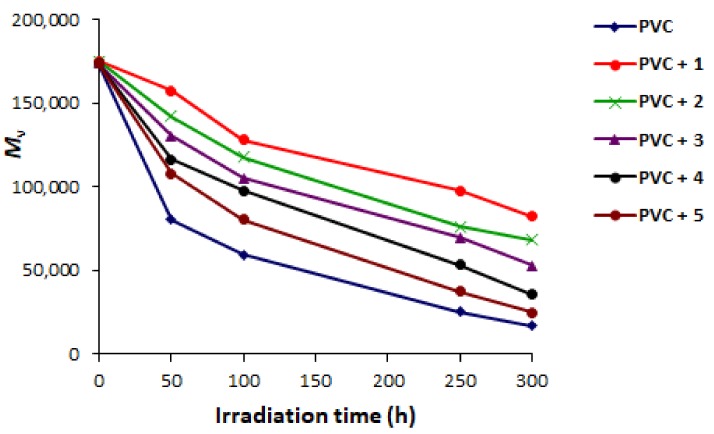
Changes in ${\bar{M}}_{V}$ for PVC.

The PVC photodegradation process leads to the formation of small polymeric fragments which are highly insoluble in THF [57]. Such fragments are responsible for the PVC cross-linking and branching. Therefore, it is expected that PVC viscosity average molecular weight (${\bar{M}}_{V}$) will be reduced when polymeric films were irradiated with UV light [8]. The changes in ${\bar{M}}_{V}$ gives an indication for the PVC chain scission [58]. Indeed, significant amount of solids were precipitated out during irradiation of PVC (blank) at 25 °C compared to the cases where additives were used. It was clear that the reduction in ${\bar{M}}_{V}$ was significantly higher in the case of PVC where no additives were used. Figure 6 shows the changes in ${\bar{M}}_{V}$ for PVC upon irradiation in THF. For example, the ${\bar{M}}_{V}$ was reduced from ca. 175,000 to 59,500 after 100 h of irradiation and reduced to only 16,600 after 300 h of irradiation. Clearly, the changes in ${\bar{M}}_{V}$ was minimal when Schiff base **1** was used as a photostabilizer. The ${\bar{M}}_{V}$ was reduced to ca. 128,200 and then to 82,500 after 100 and 300 h of irradiation, respectively. Schiff base **1** has high aromatic content and contains hydroxyl group and photostabilizer PVC to a significant level. Schiff base **2** which contains carboxyl group was next to Schiff base **1** in stabilizing PVC chains.

There is an inverse relationship between the number of average chain scission (S) and ${\bar{M}}_{V}$ as shown in Equation (1) [59]. The S value depends on the ${\bar{M}}_{V}$ measured at the initial (0) and final time (t) of irradiation:

(1) $$S={\bar{M}}_{V,O\;}/{\bar{M}}_{V,t}\;-1$$

**Figure 7:**
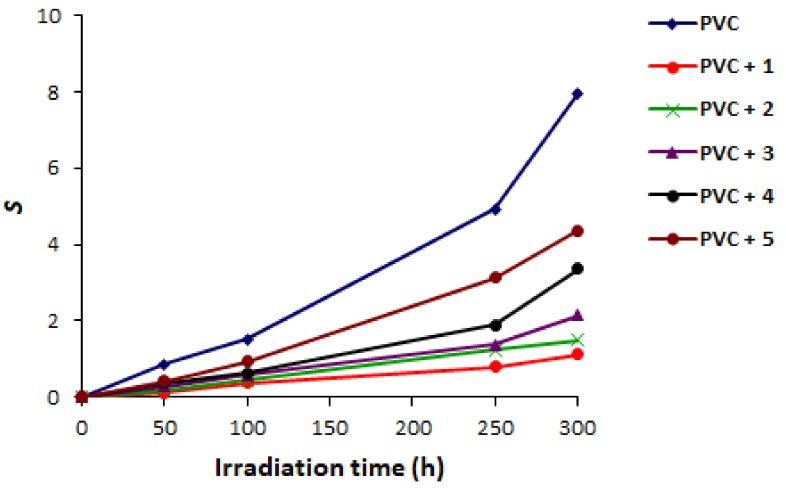
Changes in S for PVC.

Therefore, it was expected that S value can prove the degree of cross-linking and branching within the polymeric chains as a results of PVC photodegradation. Evidently, Figure 7 shows that the degree of cross-linking within PVC chains was lower when additives were used compared to the blank PVC. The minimal cross-linking degree was observed when Schiff base **1** was used. Schiff bases **2** and **3** were the most efficient additives next to Schiff base **1**.

Additionally, there is an inverse relationship between the degree of deterioration (α) and ${\bar{M}}_{V}$ as shown in Equation (2) in which m is the PVC initial molecular weight. Therefore, the value α can give an indication for the breakdown of weak bonds that are randomly distributed within the polymer backbone [60,61]:

(2) $$\alpha =m.S/{\bar{M}}_{V}$$

**Figure 8:**
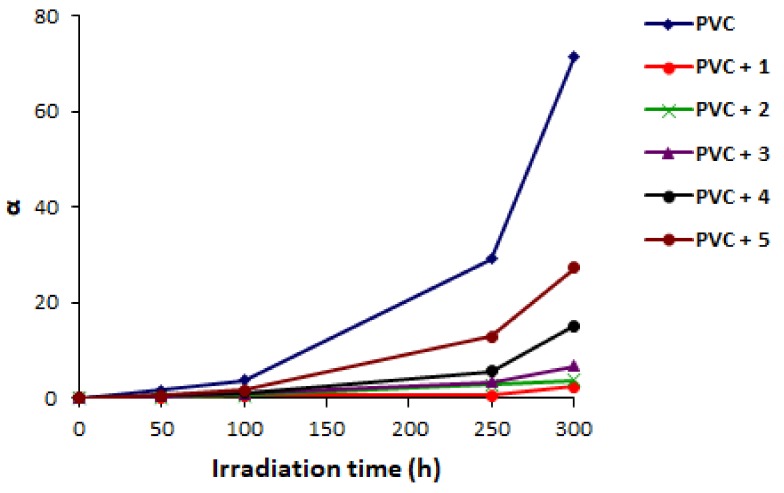
Changes in α for PVC.

Clearly, Figure 8 shows that the increases in α values was not significant in the first 100 h of irradiation. The changes in α values was very sharp after 250 h of irradiation. There was a sudden increase in the α value in the last 50 h of irradiation (between 250 and 300 h). The changes in α values were low when Schiff bases were used compared to the blank PVC. Additive **1**, which has high aromatic content, was the best additive among the ones used in terms of enhancing photostability of PVC. While, additive **5**, which contains two methyl and a carbonyl group, showed the least performance in terms of PVC photostability. The efficiency order of Schiff bases used as PVC photostabilizers follow the order **1** > **2** > **3** > **4** > **5**.

The degree of PVC photodegradation can be measured from the quantum yield of the chain scission (Φ_cs_). Equation (3) was used to calculate Φ_cs_ for PVC films after irradiation and reported in Table 4. The values of Φ_cs_ are affected by concentration (C), Avogadro’s number (A), ${\bar{M}}_{V,O}$, incident intensity (*I*_o_) and the irradiation time (*t*) in seconds:

(3) ΦCS=(CA/M¯V,O)([ηo]/[η])1α−1/Iot

The Φ_cs_ was very high (3.09 × 10^–8^) for blank PVC compared to those obtained for PVC in the presence of Schiff bases 1–5. The energy absorbed by blank PVC leads to an electronic excitation that has been distributed over a small of bonds. Therefore, such energy causes harm to the polymer chains, while, in the presence of photostabilizers. limited number of bonds have been cleaved possibly due to the involvement of many bonds at the electronic excitation sites within the PVC chains. Such process leads to a dissipation of the energy absorbed at a harmless level to PVC chains and, therefore, reduces its photodegradation [29].

### 2.4. PVC Photodegradation by Surface Morphology

**Figure 9:**
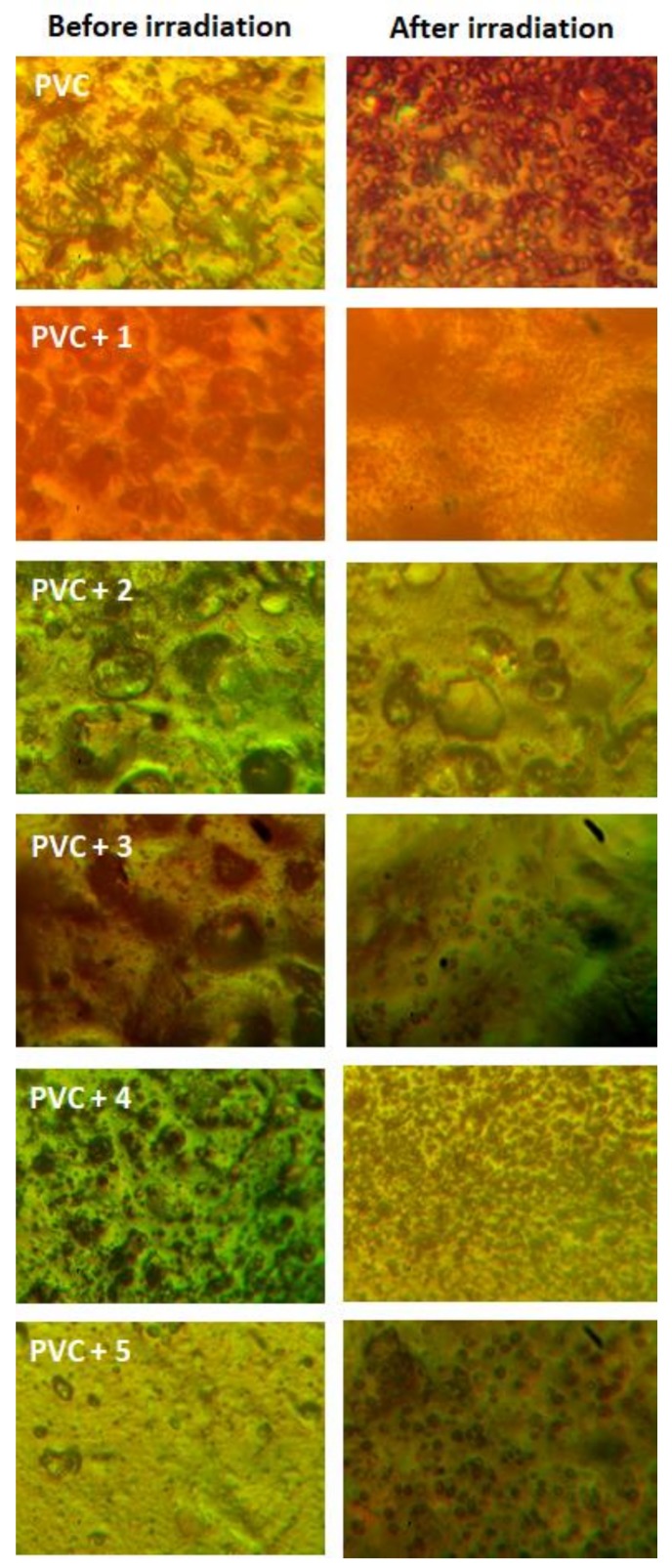
Microscopic images (400× magnification) for PVC.

To test the damages within the PVC chains, we decided to investigate the morphology of the polymeric materials after irradiation. The surface images of PVC can be used as evidence for the presence of cracks, defects and irregularity within the surface of PVC as a result of irradiation [62,63]. Figure 9 shows the surface images of polymeric films before and after irradiation (300 h). Generally, the non-irradiated PVC films showed no white spots and have smooth surfaces regardless the type of additive used. On the other hand, the images of the surface for irradiated films showed damages in terms of cracks and white spots along with the appearance of holes. However, such damages were very noticeable in the case of the blank PVC compared to the ones containing additives. Again, Schiff base **1** was very effective in stabilizing PVC, possibly through inhibition of dehydrochlorination process and, therefore, prevents elimination of hydrogen chloride from polymeric chains.

### 2.5. PVC Photostabilization Mechanisms

**Figure 10:**
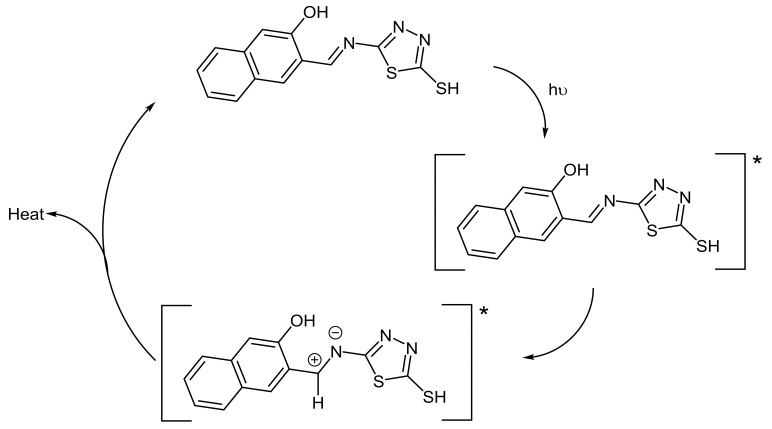
PVC photostabilization through direct absorption of radiation in the presence of **1**.

Schiff bases **1**–**5** were found to be effective against PVC photodegradation in which **1** was the most effective one. Such PVC photostability in the presence of additives could take place through various routes [64]. For example, the additives could absorb the irradiation directly in which energy can be released as a heat at a harmless level to PVC chains (Figure 10).

**Figure 11:**
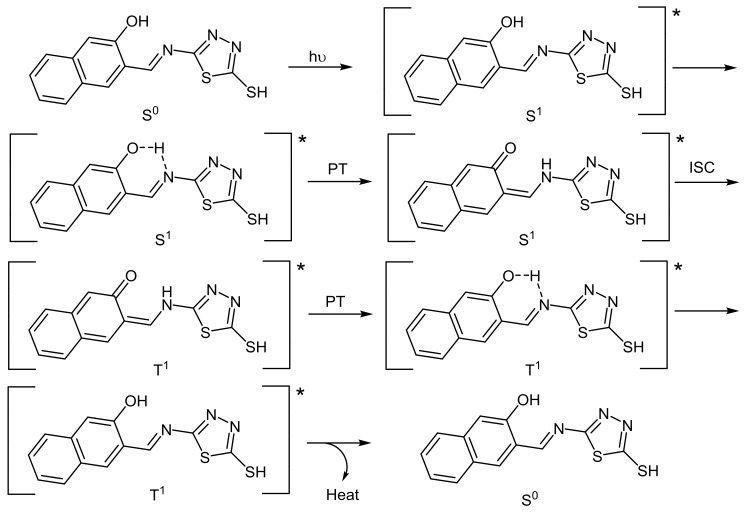
PVC photostabilization through PT and ISC in the presence of **1**.

The efficiency of Schiff bases used to stabilize PVC was found to be dependent on the type of functional group and degree of aromaticity. Schiff base 1 performed better than all other additives used. Schiff base **1** contains a hydroxyl group which plays an important role in PVC photostabilization. The presence of hydroxyl group facilitates the proton transfer (PT) and intersystem conversion (ISC) between excited states (Figure 11) [65,66]. Such processes lead to the excited energy dissipation to a harmless heat and inhibit PVC photodegradation.

**Figure 12:**
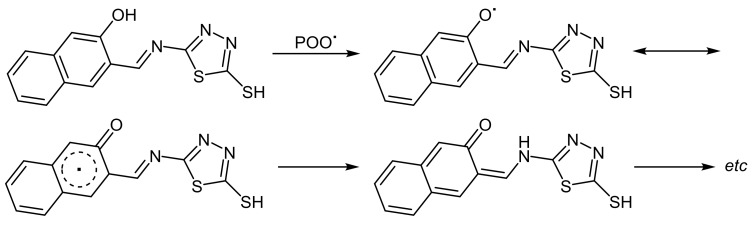
PVC photostabilization through a radical scavenger in the presence of **1**.

Additionally, the hydroxyl group in **1** could act as efficient radical scavenger [67] to provide stabilization for PVC polymeric chains against UV irradiation (Figure 12).

In addition, thiadiazole ring in Schiff bases **1**–**5** could stabilize PVC against irradiation through direct absorption of harmful UV radiation. Moreover, photostabilization could take place through the interaction between PVC and additives through coordination between carbons of carbon-chlorine bonds and lone pair of electrons on heteroatoms (e.g., oxygen, nitrogen, and sulfur) of the additives [68].

## 3. Experimental

### 3.1. General

Chemicals were obtained from Sigma-Aldrich Chemical Company (Gillingham, UK). PVC with a degree of polymerization of 800 and a K-value of 67 was obtained from Petkim Petrokimya (Istanbul, Turkey). The thickness of PVC films was adjusted using a Digital Vernier Caliper 2610A micrometer (Vogel GmbH, Kevelaer, Germany). The PVC was irradiated with a UV light at a wavelength of 250–380 nm and light intensity at 6.2 × 10^−9^ ein·dm^−3^·s^−1^ using an accelerated weather-meter QUV tester (Philips, Saarbrücken, Germany). The tester was equipped with UV-B 313 lamps to irradiate the PVC films at 25 °C. The morphology images for the PVC surface were captured using Meiji Techno microscope (Tokyo, Japan). The FTIR spectra were recorded using a Shimadzu FTIR 8000 Series spectrophotometer (400–4000 cm^−1^; Pfungstadt, Germany). A Shimadzu-160 spectrophotometer (200–900 nm; Kyoto, Japan) was used to measure the electronic spectra in dimethylformamide (1 × 10^–3^ M). Varian Mercury 300 spectrometer (Bruker, Zürich, Switzerland) was used to record the proton nuclear magnetic resonance (^1^H-NMR; 300 MHz) spectra. Mass spectra were recorded using GCMS-Q1000EX Shimadzu (70 eV; Kyoto, Japan). The Ostwald U-Tube viscometer (Ambala, Haryana, India) was used to measure the PVC viscosity.

### 3.2. Synthesis of Schiff Bases

Schiff bases **1**–**5** (Figure 2) was obtained using a reported procedure [51] and their structures were confirmed by IR, UV, ^1^H-NMR, and mass spectra.

### 3.3. PVC Films Preparation

A mixture of PVC (10 g) and Schiff bases **1**–**5** (50 mg) in THF (100 mL) was stirred for 30 min at 25 °C and then poured into glass plates. The mixture was left at room temperature for 24 h before the films were fixed (40 µm thickness) [69].

### 3.4. PVC Photodegradation by FTIR Spectrophotometry

PVC photo-oxidation of PVC produces ketones, alkene, and alcohol moieties [70,71]. Therefore, the intensity of absorption bands for the carbonyl (1724 cm^–1^), alkene (1631 cm^–1^), and hydroxyl (3400 cm^–1^) groups were monitored in the FTIR spectra and compared a reference peak (1328 cm^–1^) [53]. The indices (*I*s) for various functional groups were calculated from the absorptions of reference (*Ar*) and each functional group (*As*) using Equation (4):

(4) Is=As/Ar

### 3.5. PVC Photodegradation by Viscometry

Mark-Houwink relation was used to calculate the relative molecular weight of PVC [72]. Equation (5) was used to calculate the intrinsic viscosity, [η], was calculated using Equation (5). The [η] depends on the average molecular weight (${\bar{M}}_{V}^{\alpha }$) and constants α and K:

(5) $$\left[\eta \right]=K{\bar{M}}_{V}^{\alpha }$$

## 4. Conclusions

Five Schiff bases containing 1,3,4-thiadiazole ring systems have been used successfully at low concentrations as effective photostabilizers of poly(vinyl chloride). The additive containing 2-naphthaol was found to be the most effective. Such an additive can act as an efficient radical scavenger and ultraviolet radiation absorber, due to the presence of a hydroxyl group and its high aromatic content, respectively.

## Figures and Tables

**Table 1:** Some IR and UV spectral data for Schiff bases **1**–**5**.

**Additive**	**FR-IR (υ, cm^−1^)**					**Absorption (nm)**	
	OH/NH	SH	C=O	CH=N	C-S	π–π*	n–π*
**1**	3318	2588	—	1618	621	274, 331	383, 484
**2**	3124	2604	1786	1610	638	304	459
**3**	—	2584	—	1608	644	304	347
**4**	3178	2592	—	1610	630	279	434
**5**	—	2564	1685	1597	634	307	416

**Table 2:** ^1^H-NMR spectral data for Schiff bases **1**–**5**.

Additive	^1^H-NMR (400 MHz: DMSO-*d*_6_, δ, ppm, *J* in Hz)
**1**	12.97 (s, exch, 1 H), 9.41 (s, 1 H), 8.80 (d, *J* = 8.1 Hz, 1 H), 8.10 (d, *J* = 8.1 Hz, 1 H), 7.89 (d, *J* = 8.1 Hz, 1 H), 7.63 (t, *J* = 8.1 Hz, 1 H), 7.44 (t, *J* = 8.1 Hz, 1 H), 7.25 (d, *J* = 8.1 Hz, 1 H)
**2**	13.68 (s, exch, 1 H), 9.03 (d, *J* = 8.9 Hz, 1 H), 7.91 (s, 1 H), 7.86 (t, *J* = 8.9 Hz, 1 H), 7.75 (d, *J* = 8.9 Hz, 1 H), 6.97 (d, *J* = 8.9 Hz, 1 H)
**3**	13.01 (s, exch, 1 H), 7.62 (m, 1 H), 7.56 (m, 1 H), 7.17 (m, 1 H), 2.51 (s, 3 H)
**4**	14.30 (s, exch, 1 H), 9.90 (s, 1 H), 8.49 (s, exch, 1 H), 7.79–7.73 (m, 3 H), 7.52 (d, *J* = 7.9 Hz, 2 H)
**5**	12.61 (exch, s, 1 H), 8.22 (s, 1 H), 7.58–7.51 (m, 3 H), 7.45 (d, *J* = 8.0 Hz, 2 H), 3.38 (s, 3 H), 2.86 (s, 3 H)

**Table 3:** Mass spectral data for Schiff bases **1**–**5**.

Additive	MS (*m/z*; %)
**1**	288 ([M + 1]^+^, 17), 287 (M^+^, 100), 270 (30), 254 (32), 211 (64), 196 (33), 182 (35), 169 (44), 154 (23), 127 (96), 115 (35), 77 (30)
**2**	266 ([M + 1]^+^, 12), 265 (M^+^, 90), 220 (5), 133 (100), 105 (20), 77 (22)
**3**	242 ([M + 1]^+^, 22), 241 (M^+^, 61), 227 (38), 133 (100), 74 (22), 57 (50)
**4**	288 ([M + 1]^+^, 18), 287 (M^+^, 100), 255 (12), 240 (10), 182 (36), 155 (39), 128 (33), 77 (73), 57 (70)
**5**	332 ([M + 1]^+^, 16), 331 (M^+^, 94), 313 (10), 273 (15), 258 (20), 226 (21), 214 (35), 183 (15), 133 (47), 77 (61), 56 (100)

**Table 4:** The Φ_cs_ for PVC films after irradiation.

PVC Film	Φ_cs_
PVC	3.09 × 10^–^^8^
PVC + **1**	8.82 × 10^–^^10^
PVC + **2**	1.42 × 10^–^^9^
PVC + **3**	2.65 × 10^–^^9^
PVC + **4**	6.29 × 10^–^^9^
PVC + **5**	1.35 × 10^–^^8^
